# Early warning signs for tipping points in systems with non-Gaussian $\alpha$-stable noise

**DOI:** 10.1038/s41598-025-88659-0

**Published:** 2025-04-21

**Authors:** Lucia S. Layritz, Anja Rammig, Ilya Pavlyukevich, Christian Kuehn

**Affiliations:** 1 School of Life Science, Technical University of Munich, Hans-Carl-v.-Carlowitz-Platz 2, Munich, 85354 Germany; 2 Institute of Mathematics, Friedrich Schiller University Jena, Ernst-Abbe-Platz 2, Jena, 07743 Germany; 3 Department of Mathematics, Technical University of Munich, Boltzmannstrasse 3, Garching, 85748 Germany

**Keywords:** Tipping point, Early warning, Stochastic dynamical system, Global change, Climate sciences, Ecology, Environmental sciences, Natural hazards, Mathematics and computing, Physics

## Abstract

Forecasting rapid, non-linear change or so-called tipping points is a major concern in ecology and environmental science. Statistical early warning signs, based on the theory of stochastic dynamical systems, are now regularly applied to observational data streams. However, the reliability of these early warning signs relies on a number of key mathematical assumptions, most notably the presence of Gaussian noise, while many ecological systems exhibit non-Gaussianity. We here show that for systems driven by non-Gaussian, $\alpha$-stable noise, the classical early warning signs of rising variance and autocorrelation are not supported by mathematical theory, and their use poses the danger of spurious, false-positive results. To address this, we provide a generalized approach by introducing the scaling factor $\gamma _X$ as an alternative early warning sign. We show that in the case of the linear Ornstein-Uhlenbeck process, there exists a direct inverse relationship between $\gamma _{X}$ and the bifurcation parameter, telling us that $\gamma _{X}$ will increase as we approach the bifurcation. Our numerical simulations confirm theoretical results and show that our findings generalize well to non-linear, non-equilibrium systems often employed in ecological systems. We thus provide a generalized, robust, and broadly applicable statistical early warning sign for systems driven by Gaussian and non-Gaussian $\alpha$-stable noise.

## Introduction

Non-linear dynamical systems may exhibit rapid and irreversible state shifts upon a small change of a parameter^1,2^. The existence of such critical transitions or tipping points is a major concern in the context of global climate change^3–5^ and has been postulated for a number of ecological contexts including forests^6,7^, grasslands^8,9^ and marine ecosystems^10,11^.

In the case of stochastic systems, statistical early warning signs may precede the actual tipping point, for example, a rise in variance or autocorrelation^12,13^. A range of real-world systems exhibit such signs before critical transitions^14–17^. An increase in variance and other observables has also been observed in time series data of ecosystems and climate elements suggested to approach tipping points^18–20^ and consequently postulated for biosphere monitoring^21^.

The use of variance or autocorrelation as early warning signs sits on a robust body of mathematical theory concerned with bifurcations in stochastic dynamical systems^1,2,22^. However, one key assumption of this theory is that we are working in the small noise limit of Gaussian white noise, an assumption that may not always hold in real-world applications^23^. Previous studies have already pointed out situations where classical early warning signs fail for other noise types^24–26^.

There is ample evidence that the assumption of Gaussian white (that is uncorrelated) noise does not hold for many environmental variables which have been shown to be correlated in time^27–30^ or exhibit heavy tails, thus violating the Gaussian assumption^28,31^. Since climate change is expected to lead to a higher frequency of extreme events^32,33^, the occurrence of heavy-tailed data might additionally become more frequent in the future.

One class of probability distributions that are characterized by such heavy tails are $\alpha$-stable distributions^28,34,35^. The exception to this rule is the Gaussian (Normal) distribution which is a special case. Other known members of the class include the Cauchy (Lorenz) distribution or the Lévy distribution. A range of real-world systems have been found to display $\alpha$-stable properties^36–39^. Notable examples in ecology include the foraging behavior of various animal populations^40–43^, tree ring^44^ or the distribution of rainfall, temperature, and other climatological variables,^31,45,46^.

One important characteristic of non-Gaussian $\alpha$-stable distributions is that their variance and higher order moments diverge^35^. This has spawned much discussion about the applicability of $\alpha$-stable models to real data, as empirical moments will of course always be finite^31,46^. However, as^31^ among others have pointed out, divergence simply means that we cannot expect moments to converge to a finite value but must rather assume them to continue increasing with sample size. This of course heavily challenges the use of a rising variance as an early warning sign of a tipping point. While the use of $\alpha$-stable noise in modeling tipping points is gaining traction^47–52^, the impact of $\alpha$-stable driving noise on the existence and properties of early warning sign in such systems has not yet been assessed.

In this paper, we want to discuss the applicability and limits of classical early warning signs in the $\alpha$-stable case, as well as the implications for ecological research. In “Theoretical background” section, we revise the basic theory of stochastic dynamical systems, $\alpha$-stable processes, and early warning signs and discuss potential pitfalls when applying classical early warning signs to systems driven by $\alpha$-stable noise. In “An early warning indicator for non-Gaussian*α*-stable systems” section, we introduce an alternative early warning sign - the scaling factor $\gamma$ - showing that it is a natural generalization of the Gaussian variance scaling to the $\alpha$-stable case. Lastly, in “Numerical simulations” section, we demonstrate the applicability of our generalized approach for simple numerical models: a linear system of Ornstein-Uhlenbeck type and a non-linear system passing through a fold bifurcation, as found in many ecological models. We conclude by discussing the implications of our results for more ecological studies of tipping points.

## Theoretical background

### Stochastic dynamical systems

The application of stochastic dynamical systems has a long history in ecological and environmental science^53,54^. The study of tipping points, in particular, also references seminal works by^29^ and others that separated the slow dynamics of climate from the fast fluctuations of weather, represented by noise. In all these cases, observations are produced by the interaction of the dynamical system with the driving noise. We can formulate this view as a one-dimensional stochastic differential model

1 $$\begin{aligned} dX(t) = -U'\big (X(t), k \big )\, dt + dN(t), \end{aligned}$$

where $dX(t) = -U'\big (X (t), k \big )\, dt$ describes a deterministic dynamical system evolving in a potential *U*, *dN* stands for external noise, *X*(*t*) is a realization of the system at time *t*, and *k* is a bifurcation parameter.

The potential $U=U(X,k)$ can be chosen to represent any dynamical model suitable for the research task at hand. In this paper, we will consider two models: The (linear) Ornstein–Uhlenbeck with

2 $$\begin{aligned} U(X,k) = k\frac{X^2}{2} \quad \text {and} \quad dX(t) = -kX(t)\,dt \end{aligned}$$

as well as a non-linear, quadratic system with

3 $$\begin{aligned} U(X,k) = \frac{X^3}{3} - kX \quad \text {and} \quad dX(t) = (k - X(t)^2)\,dt. \end{aligned}$$

**Fig. 1:**
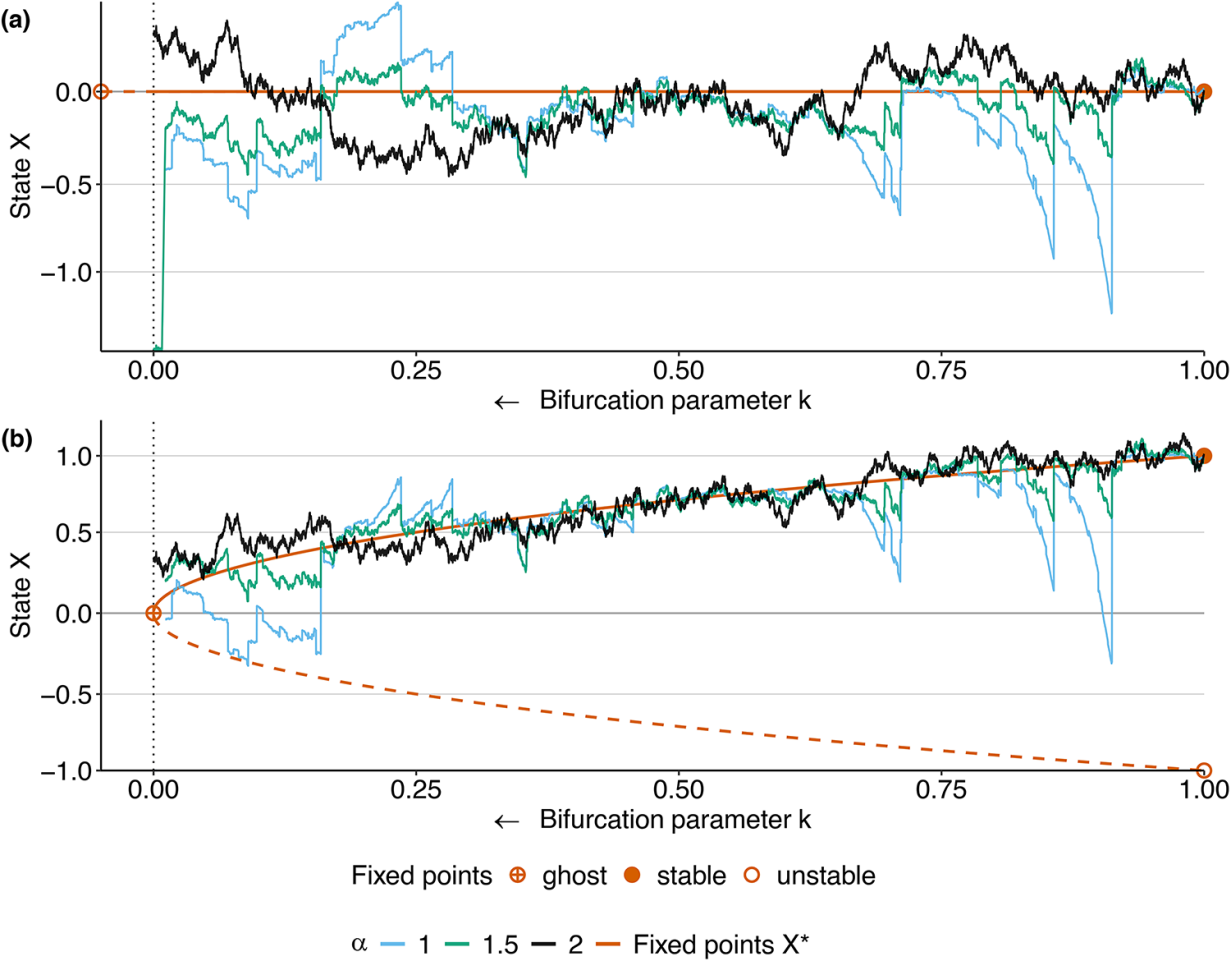
Bifurcation diagrams (orange lines) and example trajectories of the Ornstein-Uhlenbeck process (2) (**a**) and the fold bifurcation (3) (**b**). Stable fixed points are indicated by solid, unstable ones by dashed lines. Example trajectories for different values of $\alpha _N$ are obtained by slowly varying *k* in the direction of the bifurcation at $k = 0$ while following the stable fixed point. All noise sequences are generated with the same random seed to ensure extreme values occur simultaneously.

In the linear case, the deterministic dynamical system has one fixed point at $X^* = 0$. It passes through a bifurcation at $k = 0$, where $X^*$ is stable for $k > 0$ and unstable for $k < 0$ (see orange lines in Fig. 1a for a bifurcation diagram).

The linear stochastic system

4 $$\begin{aligned} dX(t) = -kX(t)\,dt + dN(t) \end{aligned}$$

gives rise to the so-called Ornstein–Uhlenbeck type process that originally described the movement of a particle subject to the random influence of the surrounding fluid and viscous friction^55^. Due to this, it is common to refer to the dynamical part as the *drift term* and the stochastic part as the *diffusion term*. The Ornstein-Uhlenbeck process is the most basic stochastic dynamical system and can be recovered from non-linear systems when linearizing around fixed points (as demonstrated in (15)).

The nonlinear system with the potential (3) has a fold bifurcation, also at $k=0$. It has two fixed points $X^*_{\pm } = \pm \sqrt{k}$ for $k > 0$ that coincide for $k=0$, and none for $k < 0$. (see orange lines in Fig. 1b for a bifurcation diagram).

The second important modeling choice to make is that of the random noise perturbation. Usually, the noise is assumed to be produced by a Brownian motion, which is a process with independent and stationary increments following a Normal (Gaussian) distribution (As we will discuss in the next chapter in more detail, this is an $\alpha$-stable process with $\alpha$ = 2). Figure 1 gives example trajectories of such systems in black.

It is evident that the choice of noise *dN* is decisive for all the stochastic properties of the process *X*. In this paper, we want to go beyond the Gaussian paradigm and focus on the case of noise *dN* that is produced by a symmetric $\alpha$-stable process $N^{(\alpha )}$, that has stationary, independent increments, but whose increments $N^{(\alpha )}(t) - N^{(\alpha )}(s)$ follow a symmetric $\alpha$-stable (Lévy) distribution. The wide class of $\alpha$-stable distributions appears naturally in the framework of a generalized Central Limit Theorem as a scaled limit of sums of independent random variables with infinite second moments. It includes the Gaussian as well as a range of heavy-tailed distributions.

In modeling, $\alpha$-stable distributions appear naturally as a “second best” choice noise that allows describing large (catastrophic) jumps or heavy tails that are not possible in “Gaussian” systems. Notable examples of non-Gaussian ecological models include the foraging behavior of animals^40–43^ or forest disturbances^49^.

### $\alpha$-stable noise

The goal of this section is to briefly revise the most important properties of symmetric centered $\alpha$-stable processes needed for our results ($\alpha$-stable processes are a subclass of Lévy processes^56,57^. For this reason, the name *Lévy stable* process is also sometimes used^34,58^. *Lévy flights* describe random walks following an $\alpha$-stable random variable^34,39^.) We will follow the notation of Section 1 in^35^ and say that a random variable *Z* has a symmetric $\alpha$-stable distribution, $Z^{(\alpha )}\sim \textbf{S}(\alpha , \beta , \gamma , \delta )$ described by four parameters:

- a characteristic exponent $\alpha \in (0,2]$, describing the tail behavior of *Z*;
- a scale parameter $$\gamma \ge 0$$;
- a skewness parameter $\beta \in [-1,~1]$, with $\beta = 0$ in the symmetric case;
- a location parameter $\delta \in \mathbb {R}$, with $\delta = 0$ in the centered case.

For the purpose of this study, we will focus on the symmetric centered $\alpha$-stable processes $\textbf{S}\alpha \textbf{S}(\gamma )$, where $\beta , \delta$ = 0. Its characteristic function is

5 $$\begin{aligned} \mathbb E e^{iuZ^{(\alpha )}}=e^{-\gamma ^\alpha |u|^\alpha },\quad u\in \mathbb R,\quad \alpha \in (0,2]. \end{aligned}$$

Despite the very simple form of the characteristic function (5), the probability density functions of $\alpha$-stable random variables are, in general, not available. There are two notable exceptions to this:

For $\alpha =2$, the random variable $Z^{(\alpha )}$ is Gaussian with the probability density function

6 $$\begin{aligned} f_{2,\gamma }(x) = \frac{1}{2\gamma \sqrt{\pi }} e^{-{\frac{x^2}{4\gamma ^2}}},\quad x\in \mathbb R. \end{aligned}$$

In this case,

7 $$\begin{aligned} \mathbb {E}[Z^{(2)}]=0 \quad \text {and} \quad \operatorname {Var} Z^{(2)}=2\gamma ^2. \end{aligned}$$

For $\alpha =1$, the random variable $Z^{(\alpha )} \sim \textbf{S}\alpha \textbf{S}(\gamma )$ has the Cauchy (Lorenz) distribution with the density

8 $$\begin{aligned} f_{1,\gamma }(x) = \frac{\gamma }{\pi (\gamma ^2+x^2)},\quad x\in \mathbb R \end{aligned}$$

It is instructive to note that for $\alpha =1$, neither the variance nor the mean value of $Z^{(1)}$ exist.

In general, for $\alpha \in (0,2)$ and $\gamma >0$ for the random variable $Z^{(\alpha )}$ we have:

9 $$\begin{aligned} \mathbb {E} [|Z^{(\alpha )}|]^\eta ={\left\{ \begin{array}{ll} <\infty ,\quad \eta \in (0,\alpha ), \\ +\infty ,\quad \eta \in [\alpha ,\infty ). \end{array}\right. } \end{aligned}$$

In the context of statistical early warning signs, it is important to note that the second moment and variance are not defined for all $\alpha \in (0,2)$, and the first moment (mean) is not defined for all $\alpha \in (0,1]$. On the other hand, all moments exist in the Gaussian case $\alpha =2$.

With the definition of a $\textbf{S}\alpha \textbf{S}(\gamma )$ random variable in hand, we can construct a process $N^{(\alpha )}$ starting at zero such that its increments

10 $$\begin{aligned} N^{(\alpha )}(t_1),\ N^{(\alpha )}(t_2)-N^{(\alpha )}(t_1),\ \dots ,\ N^{(\alpha )}(t_n)-N^{(\alpha )}(t_{n-1}) \end{aligned}$$

are independent for any choice of $n\ge 1$ and $0\le t_1\le \cdots \le t_n$ (the white noise property) and

11 $$\begin{aligned} N^{(\alpha )}(t)-N^{(\alpha )}(s)\sim \textbf{S}\alpha \textbf{S}(\gamma _N(t-s)^{1/\alpha }). \end{aligned}$$

**Fig. 2:**
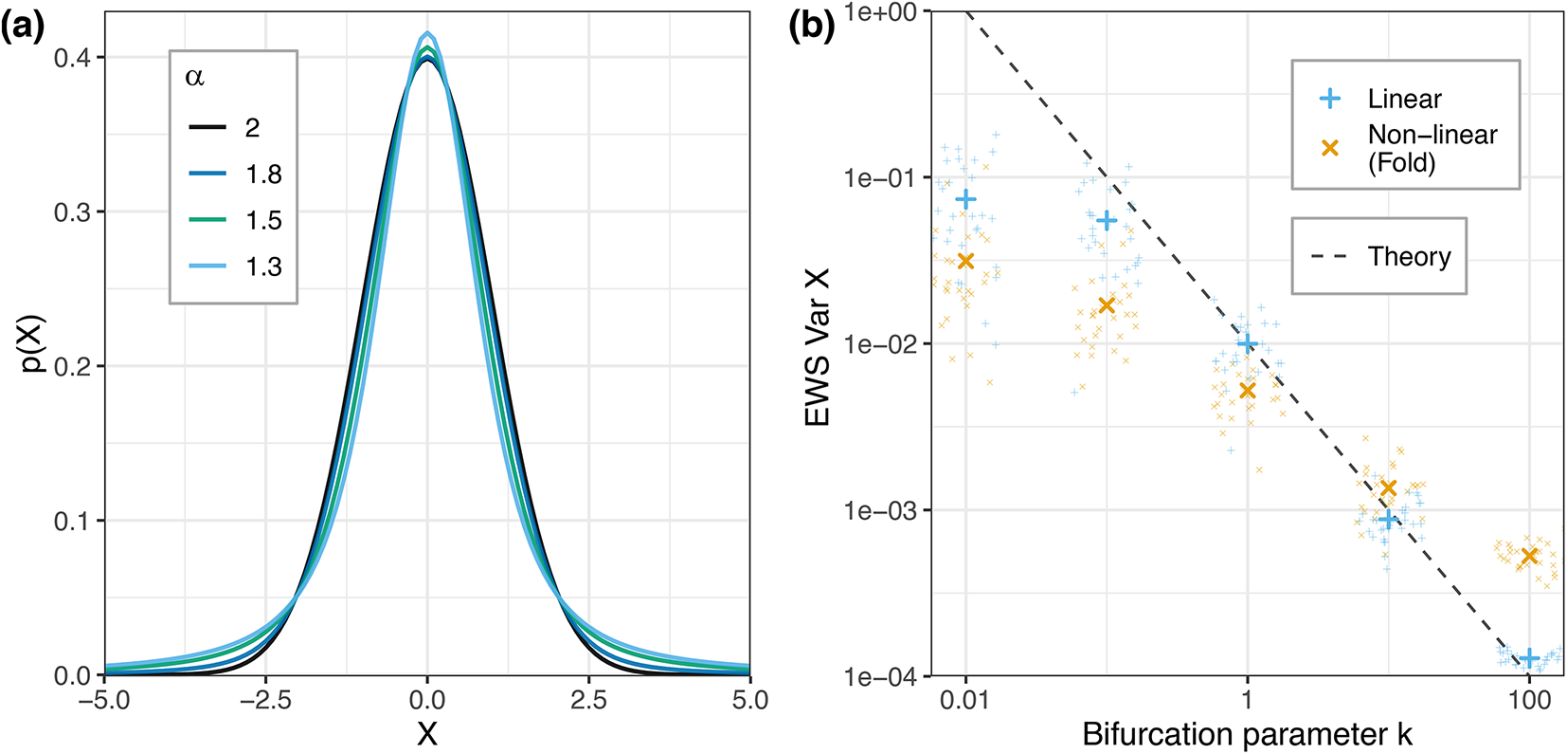
(**a**) The classical early warning sign: Relationship between bifurcation parameter *k* and $\operatorname {Var} X$ in the Gaussian case for the Ornstein-Uhlenbeck process (4) (blue) and the fold bifurcation (3) (yellow). Dashed line gives theoretical result (14). (**b**) Probability density functions of $\alpha$-stable random variables for different values of $\alpha$ ($\beta = 0$, $\gamma = 1$, $\delta = 0$).

In other words, for $\alpha =2$, the process $N^{(2)}$ is a Brownian motion that has exponentially light tails and continuous trajectories^56^. For $\alpha \in (0,2)$, the process $N^{(\alpha )}$ has heavy tails and discountinous trajectories. Figure 1b illustrates the effect of $\alpha$ on trajectories of $N^{(\alpha )}$. Figure 2a illustrates the effect of the characteristic exponent $\alpha$ on the shape of the distribution of the random variable $Z^{(\alpha )}$.

### Early warning signs

To construct early warning signs, we are interested in the statistical properties of the stochastic process *X* in relation to the bifurcation parameter *k* and the noise $N^{(\alpha )}$. We would like to reiterate that changes in these properties when approaching a bifurcation are created through the interaction of the driving noise $N^{(\alpha )}$ with the dynamical system. The driving perturbation itself is assumed to remain constant.

A range of statistical properties of *X* has been utilized as early warning signs. The most important one, which we will focus on for the remainder of this work, is variance $\operatorname {Var} X(t)$. However, autocorrelation $\zeta$^1,5,13^, skewness^59^ or other properties^60,61^ have also been proposed.

The theory of early warning signs sits on a robust body of mathematical theory derived from the properties of the Ornstein-Uhlenbeck process (4): For this particular system, we can obtain an explicit solution (following^22^)

12 $$\begin{aligned} X(t) = X_0e^{-kt} + \int _0^t e^{-k (t-s)}\, dN^{(\alpha )}(s). \end{aligned}$$

Recall that in the classical case $\alpha =2$, we assume $N^{(2)}$ to be a Brownian motion with increments $N(t) - N(s) \sim \mathcal {N}(0, 2\gamma _N^2(t-s))$. Assuming for definiteness that $X_0=0$, we obtain that *X*(*t*) will be normally distributed with mean $\mu = 0$ as well. We can obtain the full probability density *p*(*X*, *t*) from(12) directly or via the Fokker-Planck equation

13 $$\begin{aligned} \frac{\partial p(X,t)}{\partial t} = \frac{\partial }{\partial X}\Big (kX p(X, t)\Big ) +\gamma _N^2\frac{\partial ^2p(X, t)}{\partial X^2} \end{aligned}$$

to obtain the variance

14 $$\begin{aligned} \operatorname {Var} X(t) = \frac{\gamma _N^2}{2k}(1-e^{-2kt}) {\mathop {=}\limits ^{t \rightarrow \infty }} \frac{\gamma _N^2}{2k} . \end{aligned}$$

Hence we find that in the stationary regime ($t\rightarrow \infty$), the limit variance scales with $\frac{1}{2k}$ and hence increases as the system approaches the stable-to-unstable transition ($k~\rightarrow ~0^+$), as shown in Fig. 2b.

In non-linear systems, one would typically linearize around the steady state of interest to again obtain a linear system of the form of (4)^19,23^. In the case of the fold bifurcation (3) we expand the right-hand side of $f(X)=k-X^2$ around $X^*_+=\sqrt{k}$:

15 $$\begin{aligned} \begin{aligned} f(X)&= f(X^*_+) + f'(X^*_+)(X - X^*_+) + \mathcal {O}(|X -X^*_+|^2). \end{aligned} \end{aligned}$$

Rearranging the terms and denoting $Y = X - X^{*}_+$ and $\kappa = 2\sqrt{k}$ we obtain an approximation of *X* in the vicinity of $X^*_+$ as a new Ornstein–Uhlenbeck process

16 $$\begin{aligned} dY = -\kappa Y\, dt + dN^{(\alpha )}. \end{aligned}$$

Hence we can expect the system to still follow relationship (14) when close to $X^*_+$.

In the case of non-Gaussian $\alpha$-stable noise, $N^{(\alpha )}$ will not possess a finite variance, as stated in Section 2.2.^58^ show that for systems of type (1) with $\alpha$-stable driving noise $N^{(\alpha )}$ and *U*(*X*) of order $|x|^c$, $|x|\rightarrow \infty$, Var[*X*(*t*)] will only be finite if $c > 4 - \alpha$. Only then is the potential function steep enough to sufficiently confine the noise. In the case of an Ornstein–Uhlenbeck process we have $c=2$ and therefore $c > 4 - \alpha$ does not hold for $\alpha \in (0,2)$.

The same is true for the fold bifurcation system with $c=3$ and $\alpha \in (0,1]$. In the case of more complex systems such as a quartic double-well potential ($c=4$), the global variance exists. Nevertheless, when we apply linearization as in equation (15), the local existence of variance gets lost.

**Fig. 3:**
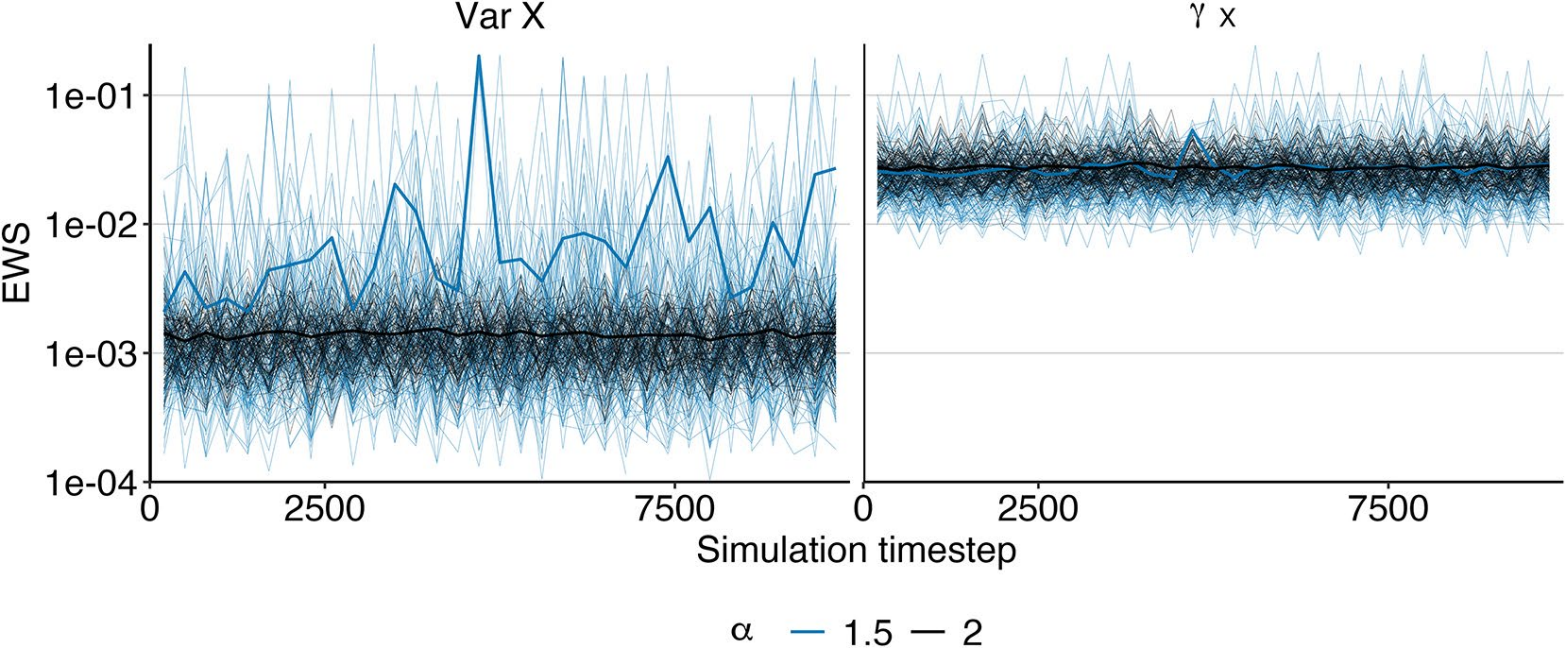
Illustration of non-converging variance. In the Gaussian case ($\alpha$ = 2), the variance converges to a final value once the simulation has reached equilibrium due to the central limit theorem (black trajectories in the left panel). In the non-Gaussian, $\alpha$-stable case, the variance of *X* does not converge to a finite value and may thus exhibit a spurious increase even for constant *k*, giving rise to a false-positive early warning sign (blue trajectories in the left panel). In contrast, $\gamma _X$ converges for all $\alpha$ (right panel). Thin lines are individually simulated trajectories, bold lines average over all 100 trajectories, *k* = 1. Note the log scale on the y-axis. Simulation setup mirrors Fig.  5, $\gamma _X$ is estimated every 300 simulation steps.

This implies that the classical theory of early warning signs relying on linearization, as laid out in this section is not valid for systems driven by non-Gaussian $\alpha$-stable noise. On the contrary, as we cannot ensure the variance to converge to a finite value, there is always the danger of misinterpreting resulting spurious increases as an early warning sign (see left panel of Fig. 3 for an illustration).

Therefore, where systems driven by non-Gaussian $\alpha$-stable noise might occur, we are in need of a different indicator that is robust again violating the Gaussian assumption.

## An early warning indicator for non-Gaussian $\alpha$-stable systems

To address this caveat, we propose the scaling parameter $\gamma _X$ as an alternative, robust early warning sign that is applicable to Gaussian and $\alpha$-stable systems, easy to calculate in practical applications, and, as we will show in the following, firmly grounded in mathematical theory.

We start with the $\alpha$-stable process of Ornstein-Uhlenbeck *type*

17 $$\begin{aligned} dX(t) = -kX(t)\,dt + dN^{(\alpha )}(t), \end{aligned}$$

where $N^{(\alpha )}(t)$ is a symmetric, $\alpha$-stable process with characteristic function

18 $$\begin{aligned} \mathbb {E}[e^{iuN^{(\alpha )}(t)}] = e^{-\gamma _{N}^{\alpha }t|u|^{\alpha }} . \end{aligned}$$

Recall the solution of the Ornstein-Uhlenbeck process (12). Assuming for simplicity that $X_0=0$, we obtain (see Appendix for a full derivation) that *X*(*t*) is also $\alpha$-stable with the characteristic function

19 $$\begin{aligned} \mathbb {E}[e^{iuX(t)}] = e^{-\gamma _{X}^{\alpha }(t)|u|^{\alpha }}, \end{aligned}$$

with the scale parameter

20 $$\begin{aligned} \gamma _{X}(t) = \gamma _N \root \alpha \of {\frac{1 - e^{-\alpha k t}}{\alpha k}},\quad t\ge 0. \end{aligned}$$

In the stationary regime $t\rightarrow \infty$ we obtain the limit value of the scale parameter

21 $$\begin{aligned} \gamma _{X}(t) = \gamma _N \root \alpha \of {\frac{1 - e^{-\alpha k t}}{\alpha k}}{\mathop {=}\limits ^{t \rightarrow \infty }} \gamma _N \root \alpha \of {\frac{1}{\alpha k}}=:\gamma _X. \end{aligned}$$

This formula gives us a direct relationship between the limit scale $\gamma _{X}$ and the bifurcation parameter *k*, which tells us that $\gamma _{X}$ will increase as we approach the bifurcation (decreasing *k*). Based on this relationship, we are able to utilize $\gamma _X$ as an early warning sign of that bifurcation.

This is indeed a generalization of the variance scaling found in the Gaussian case. Recalling the relation (7) for Gaussian $\alpha$-stable variables and taking into account (14) we get

22 $$\begin{aligned} \gamma _{X} = \gamma _N \sqrt{\frac{1}{2k}} \end{aligned}$$

recovering (21) for $$\alpha =2$$.

## Numerical simulations

We perform a range of numerical simulations to confirm our results and to illustrate the applicability of our proposed indicator $\gamma _{X}$. As (21) gives the solution in the long-term limit, we first perform *equilibrium simulations* for both systems (2) and (3) over a range of values for *k*. In a second step, we then estimate $\gamma _{X}$ from a single trajectory while slowly moving *k* towards the bifurcation, as one would in actual applications (*non-equilibrium simulations*). We perform all simulations for $\alpha$ = {2, 1.8, 1.5, 1.3}. We focus on this range, as it is what typically occurs in real and simulated applications.

To discretize and simulate the sample paths of *X*, the Euler-Maruyama scheme^45,62,63^

23 $$\begin{aligned} X_{j\Delta t} = X_{(j-1)\Delta t} - U'(X_{(j-1)\Delta t}) \Delta {t}+ \root \alpha \of {\Delta {t}}N^{(\alpha )}_{j},\quad j\ge 0, \end{aligned}$$

is often used. It is well known that for superlinear drifts $-U'$, this scheme is not stable and often results in diverging trajectories, see, e.g.^64^. In practice, such samples are often neglected, which leads to biased numerical results.

For our simulations, we thus use the *tamed* Euler–Maruyama method:

24 $$\begin{aligned} X_{j} = X_{j-1} - \frac{U'(X_{j-1})}{1 + \epsilon |U'(X_{j-1})|} \Delta {t}+ \root \alpha \of {\Delta {t}}N^{(\alpha )}_{j},\quad j\ge 0. \end{aligned}$$

For $\epsilon \ll \Delta t$ small enough, the modified drift appearing in Eq. (24) is very close to $-U'$, but remains bounded as *t* approaches infinity. This makes the numeric scheme stable with respect to the large jumps of the noise $N^{(\alpha )}$^64–66^.

We assume that ${N_j}$ are i.i.d. $\alpha$-stable random variables with the characteristic function

25 $$\begin{aligned} \mathbb {E}e^{iuN^{(\alpha )}_j} = e^{-\gamma _{N}^{\alpha }|u|^{\alpha }}. \end{aligned}$$

They are obtained with the help of the Python function

$$\begin{aligned} \texttt {stats.levy}\_\texttt {stable.rvs}(\texttt {alpha = }\alpha _{N}, \,\texttt {beta = 0},\, \texttt {loc = 0}, \,\texttt {scale} = \gamma _N). \end{aligned}$$

For our simulations, we choose $\gamma _{N} = 0.1$ and $\Delta {t} = 0.004$, and initiated all simulations at $X_0 = 0.5$, to be in the vicinity but not at the stable state. Since we have more than one fixed point in the non-linear case, trajectories might escape the basin of attraction of the stable fixed point. We therefore stop a simulation if

26 $$\begin{aligned} X_{j} < -\sqrt{k} - \frac{k}{10}. \end{aligned}$$

For the equilibrium runs we perform 100 independent estimations of $\gamma _{X}$ for each combination of $\alpha$ and *k*. As our goal here is to confirm our theoretical findings, we use 5 independent trajectories for each estimation to improve accuracy at reasonable computational costs (see Fig. 6). All parameters used in the simulations are also given in Table 1. Note that these are optimized for the range of $\alpha$ simulated and would need to be adapted to other cases accordingly. To reduce the influence of stochasticity on our estimations, we use the same noise sequence across the range of *k* within each estimation and the same random seed to generate noise sequences for different $\alpha$ (see Fig. 1 for an illustration of the latter).

For the non-equilibrium runs, we simulate 15 trajectories for each value of $\alpha$. After reaching equilibrium, we vary *k* from 5 to 0 in steps of 0.0001. We estimate $\gamma _X$ every 150 time steps, using 300 data points.

**Table 1:** Overview of simulation parameters.

Parameter	Equilibrium	Non-Equilibrium
Euler-Maruyama		
Time step $\Delta t$	0.004	0.004
Number of timesteps	10000	10000 + 50000
$\gamma _N$	0.1	0.1
$\alpha _N$	2.0, 1.8, 1.5, 1.3	2.0, 1.8, 1.5, 1.3
Estimation of ${\gamma _{\textbf{X}}}$		
Sample size	100	15
Number of data points	70$\times$5 trajectories	300
Values of *k*	100, 10, 1, 0.1, 0.01	5 to 0 by 0.0001

### Equilibrium simulations

**Fig. 4:**
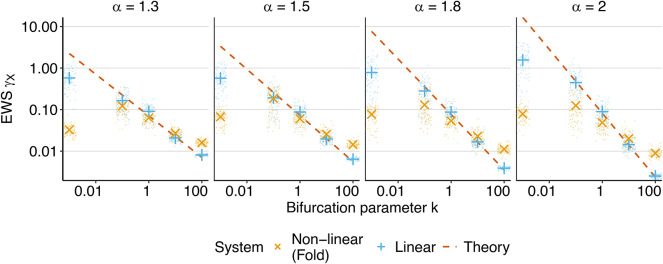
Equilibrium simulations: Estimation of $\gamma _{X}$ as a function of *k* for the (linear) Ornstein-Uhlenbeck process (4) (blue) and non-linear fold (3) (yellow) on a log-log scale. Small dots represent 100 individual estimations, large dots represent the mean value across the whole sample. Orange lines give the theoretical result (21).

Our simulations of the Ornstein-Uhlenbeck process confirm the theoretical relationship between *k* and $\gamma _X$ (Fig. 4). Accuracy is highest for large values of *k*; the smaller *k*, the higher the variability between independent estimations. However, the mean across simulations corresponds to theoretical values for all *k* and $\alpha$, only deviating slightly very close to the bifurcation.

In the non-linear case, we see similar patterns of increasing variability for lower values of *k* and $\alpha$. Mean values align with theory for medium values of *k* but not very far or very close to the bifurcation. This is expected as the linearization (15) neglects higher-order terms, which become more important as we approach the bifurcation point. Nevertheless, we observe a strong increase in $\gamma _X$ up until $k = 0.1$, confirming the theoretical suitability of $\gamma _X$ as an early warning sign across all simulated $\alpha$ for a wide range of *k*.

As expected, estimating $\gamma _X$ from trajectories produces more noisy results, with individual trajectories exhibiting large jumps in $\gamma _X$, especially for smaller $\alpha$ due to large jumps of the underlying process.

### Non-equilibrium simulations

The mean across trajectories fits the theoretical value well at the start of the simulation but begins to deviate more and more as the simulation progresses. This is consistent with theory as we are leaving the equilibrium case and the system takes longer to reach equilibrium again as we move towards a bifurcation. However, $\gamma _X$ continues to increase. An exception is the linear case for $\alpha$ values of 1.3 and 1.5, where we see a stagnation or even decline of the mean trajectory very close to the bifurcation ($k < 1$). Importantly in the non-linear case, this is not the case and we observe a steady increase in $\gamma _X$ for the whole range of *k* and all $\alpha$ in both the mean and the majority of individual trajectories. This confirms the practical suitability of $\gamma _X$ as an early warning sign of an approaching bifurcation in more application-oriented situations.

**Fig. 5:**
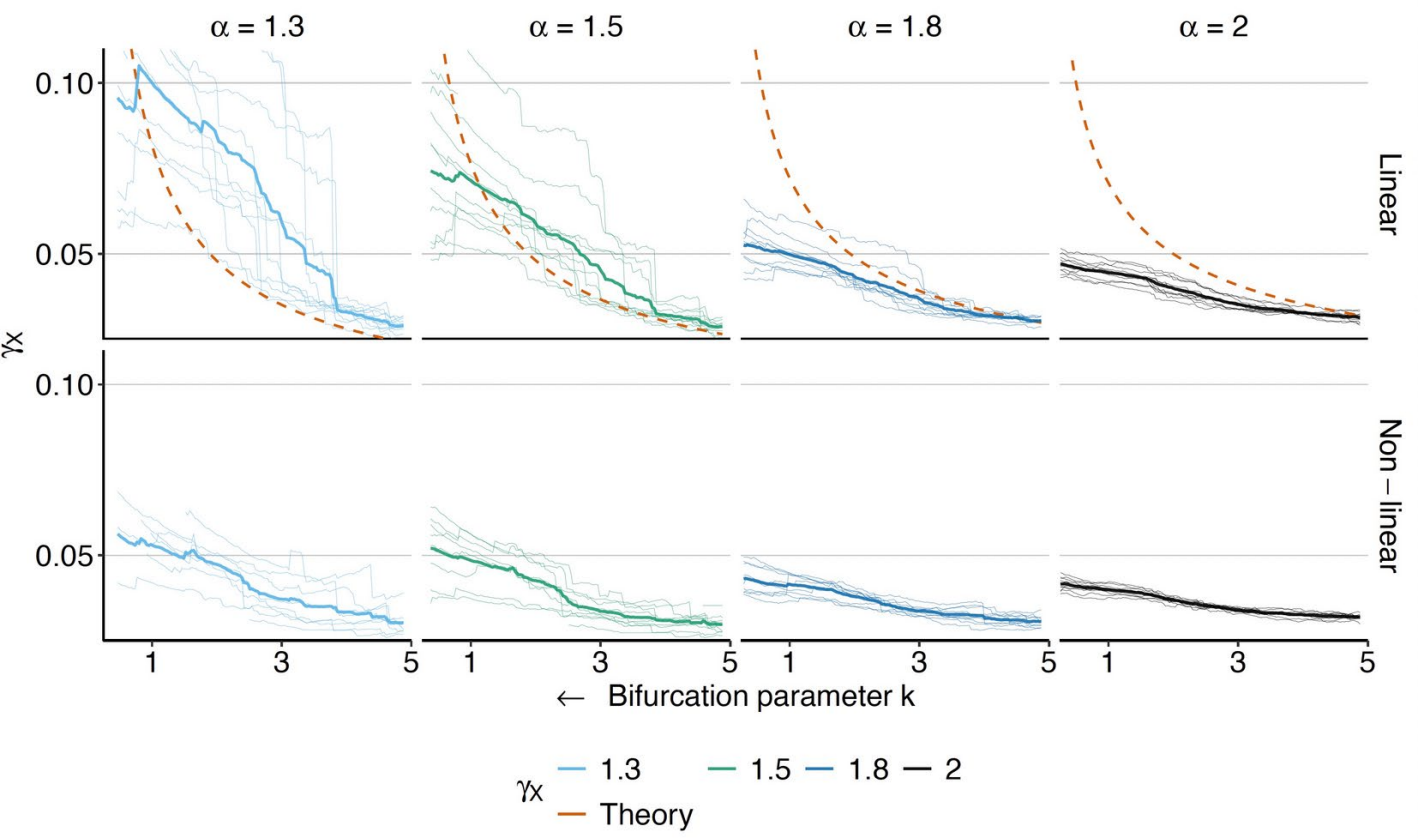
Non-equilibrium simulations: Estimation of $\gamma _{X}$ on transient trajectories produced by the linear and non-linear systems. Thin lines represent 15 individual estimations, thick lines represent the mean value across the whole sample. All trajectories are additionally smoothed using a moving window of 100 points. Dashed lines give theoretical results. *k* is moved in the direction of the arrow.

## Conclusion and implications for practical applications

We have shown that for systems driven by $\alpha$-stable, non-Gaussian noise, the classical early warning sign of rising variance is not supported by mathematical theory, and its use poses the danger of spurious, false-positive results.

To address this, we have considered the scale factor $\gamma _X$ as an alternative generalized early warning sign applicable to Gaussian and non-Gaussian $\alpha$-stable processes. We have laid out the necessary mathematical theory to show that $\gamma _X$ is always defined and inversely scales with the bifurcation parameter, much like the variance does in the Gaussian case.

Our simulations confirm our theoretical results and show that $\gamma _X$ can be estimated from few trajectories with sufficient accuracy. Additionally, our results generalize well to the non-linear, non-equilibrium case we would usually find in applications.

Our results highlight that researchers applying tipping point theory to ecological applications need to be sure that the relevant assumptions hold in their case. While the driving noise of a real ecological system might not be known — or debatably even exist — non-Gaussianty of the observable *X*(*t*) alone should caution against using metrics designed for Gaussian systems. For cases where *X*(*t*) can be described as an $\alpha$-stable random variable, we hope to have provided an alternative metric useful for practitioners.

Estimating the parameters of an $\alpha$-stable distribution is a common exercise, and algorithms are readily available in relevant programming languages. While computationally more expensive than variance estimation, it still provides an easy-to-use method that works with limited data points. This provides good conditions for applying $\gamma _X$ to more complex and real-world data streams in the future.

The utilization of statistical early warning signs has become widespread, extending beyond theoretical models to practical, policy-relevant examinations of tipping points within the climate system and their repercussions on the biosphere. Ensuring the solidity and robustness of the theoretical underpinnings supporting these applications is paramount. We thus hope our results will contribute to advancing the reliability and accuracy of early warning signs in ecological and environmental research.

## Figures and Tables

**Fig. 6:**
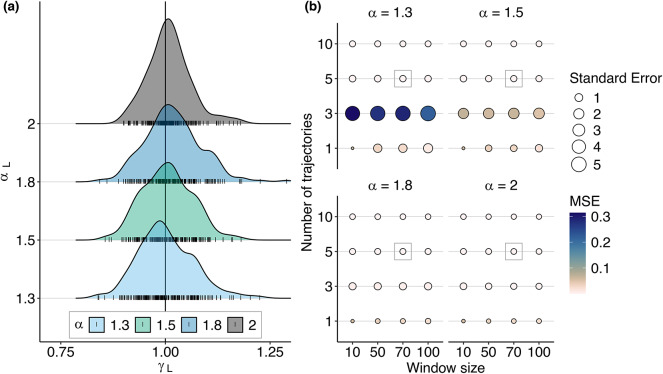
Performance of algorithm. (**a**) Accuracy of algorithm to estimate $\gamma _N$ for different values of $\alpha _N$ (True $\gamma _N = 1$, sample size is 200). (**b**) Accuracy of estimating $\gamma _X$ in dependence of window size per trajectory and number of trajectories used. Multiplying both gives total amount of data points per estimation. Color represents mean square error $\frac{| \gamma _{\text {true}} - \gamma _{\text {est}} | ^2}{\gamma _{\text {true}}}$ and size represents the standard error $\frac{\sigma _{\gamma _{\text {est}}}}{\gamma _{\text {true}}}$. Estimation time increases with number of data points (not shown). Grey box indicates parameters chosen for simulation.

## Data Availability

Code and data to reproduce all figures can be found at https://github.com/lucialayr/stableEWS_paper

## Appendix: Derivation of $\gamma _X$

We are interested in the statistical properties of the Ornstein–Uhlenbeck process *X* driven by the symmetric $\alpha$-stable process $N^{(\alpha )}$. For $t\ge 0$, let $\psi (u)$ be its characteristic function (Fig. 6),

27 $$\begin{aligned} \psi _{X(t)}(u) = \mathbb {E}[e^{iuX(t)}]. \end{aligned}$$

Using (12) with the initial condition $X_0 = 0$ we obtain

28 $$\begin{aligned} \psi _{X(t)}(u) = \mathbb {E}\exp \Big (iu\int _0^t e^{-k(t-s)}\, dN^{(\alpha )}(s)\Big ). \end{aligned}$$

Approximating the stochastic (Itô) integral by the sums over the partition $0<s_1^{(L)}<\dots <s_L^{(L)}=t$ and using the independence of increments of $N^{(\alpha )}$ we get

29 $$\begin{aligned} \psi _{X(t)}(u)&= \lim _{L\rightarrow \infty } \mathbb {E}\exp \Big (iu e^{-kt} \sum _{j=1}^L e^{ks_{j}^{(L)}} (N^{(\alpha )}(s^{(L)}_{j+1}) - N^{(\alpha )}(s^{(L)}_{j}))\Big ) \end{aligned}$$

30 $$\begin{aligned}&= \lim _{L\rightarrow \infty } \prod _{j=1}^L \mathbb {E} \exp \Big (i \underbrace{u e^{-k(t - s_{j}^{(L)})}}_{\text {u'}}(N^{(\alpha )}(s^{(L)}_{j+1}) - N^{(\alpha )}(s^{(L)}_{j}))\Big ). \end{aligned}$$

If we map the last expression to (18) and take $u' = u e^{-k(t - s_{j}^{(L)})}$ as the new Fourier parameter we obtain

31 $$\begin{aligned} \psi _{X(t)}(u)&= \lim _{L\rightarrow \infty } \prod _{j} \exp \Big ((s^{(L)}_{j+1} - s^{(L)}_{j})\gamma _{N}^{\alpha }|\underbrace{ue^{-k(t - s_{j}^{(L)})}}_\text {u'}|^{\alpha }\Big ) \end{aligned}$$

Rearranging terms and using the Itô integral again:

32 $$\begin{aligned} \psi _{X(t)}(u)&= \lim _{L\rightarrow \infty } \exp \Big (\gamma _{N}^{\alpha } \sum _{j} (s^{(L)}_{j+1} - s^{(L)}_{j}) |ue^{-\alpha k(t - s_{j}^{(L)})}|^{\alpha }\Big ) \end{aligned}$$

33 $$\begin{aligned}&= \exp \Big (-\int _0^t \gamma _{N}^{\alpha } | ue^{- k(t - s)}|^{\alpha } \, ds\Big ) \end{aligned}$$

Solving the integral, we arrive at the solution

34 $$\begin{aligned} \psi _{X(t)}(u)&= \exp \Big (-\gamma _{N}^{\alpha }|u|^\alpha e^{-\alpha kt}\int _0^t e^{\alpha ks}\, ds\Big ) \end{aligned}$$

35 $$\begin{aligned}&= \exp \Big (- \frac{\gamma _{N}^{\alpha }}{\alpha k} (1 - e^{-\alpha k t}) |u|^{\alpha }\Big ). \end{aligned}$$
